# Chemical Characterization and Evaluation of the Antibacterial Activity of Essential Oils from Fibre-Type *Cannabis sativa* L. (Hemp)

**DOI:** 10.3390/molecules24122302

**Published:** 2019-06-21

**Authors:** Ramona Iseppi, Virginia Brighenti, Manuela Licata, Antonella Lambertini, Carla Sabia, Patrizia Messi, Federica Pellati, Stefania Benvenuti

**Affiliations:** 1 Department of Life Sciences, University of Modena and Reggio Emilia, Via G. Campi 103/287, 41125 Modena, Italy; ramona.iseppi@unimore.it (R.I.); virginia.brighenti@unimore.it (V.B.); anto.lambertini@gmail.com (A.L.); carla.sabia@unimore.it (C.S.); patrizia.messi@unimore.it (P.M.); stefania.benvenuti@unimore.it (S.B.); 2 Department of Biomedical, Metabolical and Neural Sciences, University of Modena and Reggio Emilia, Via del Pozzo 71, 41124, Modena, Italy; manuela.licata@unimore.it

**Keywords:** *Cannabis sativa* L., hemp, essential oil, terpenes, cannabinoids, GC, antibacterial activity

## Abstract

Volatile terpenes represent the largest group of *Cannabis sativa* L. components and they are responsible for its aromatic properties. Even if many studies on *C. sativa* have been focused on cannabinoids, which are terpenophenolics, little research has been carried out on its volatile terpenic compounds. In the light of all the above, the present work was aimed at the chemical characterization of seventeen essential oils from different fibre-type varieties of *C. sativa* (industrial hemp or hemp) by means of GC-MS and GC-FID techniques. In total, 71 compounds were identified, and the semi-quantitative analysis revealed that α- and β-pinene, β-myrcene and β-caryophyllene are the major components in all the essential oils analysed. In addition, a GC-MS method was developed here for the first time, and it was applied to quantify cannabinoids in the essential oils. The antibacterial activity of hemp essential oils against some pathogenic and spoilage microorganisms isolated from food and food processing environment was also determined. The inhibitory effects of the essential oils were evaluated by both the agar well diffusion assay and the minimum inhibitory concentration (MIC) evaluation. By using the agar diffusion method and considering the zone of inhibition, it was possible to preliminarily verify the inhibitory activity on most of the examined strains. The results showed a good antibacterial activity of six hemp essential oils against the Gram-positive bacteria, thus suggesting that hemp essential oil can inhibit or reduce bacterial proliferation and it can be a valid support to reduce microorganism contamination, especially in the food processing field.

## 1. Introduction

**Figure 1:**
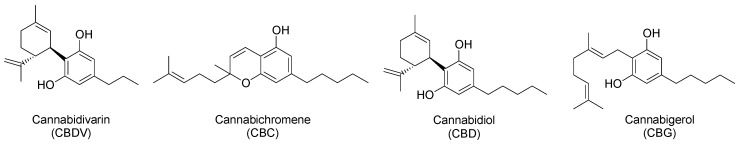
Chemical structures of hemp non-psychoactive cannabinoids.

Scientific research on fibre-type *Canabis sativa* L. (commonly known as industrial hemp or hemp) has been significantly increasing in recent years [1,2]. The wide pharmacological profile of its non-psychoactive cannabinoids, which belong to the class of terpenophenolics, make them the leading actors of the vast majority of the scientific papers related to this variety [3,4,5,6,7]. Among these compounds, cannabidiol (CBD) represents the most promising one from a pharmaceutical point of view (Figure 1). Indeed, it has displayed antioxidant, anti-inflammatory, antibacterial, anti-proliferative and neuroprotective properties [1,2,7,8,9,10,11,12]. Together with its homologue cannabidivarin (CBDV), CBD is also recognized as an effective anticonvulsant agent [13]. Cannabigerol (CBG) and cannabichromene (CBC) are other cannabinoids which can be found in hemp female inflorescences (Figure 1), and are characterized by a remarkable antibacterial activity, together with anti-inflammatory and anti-proliferative properties [1,2,12].

**Figure 2:**
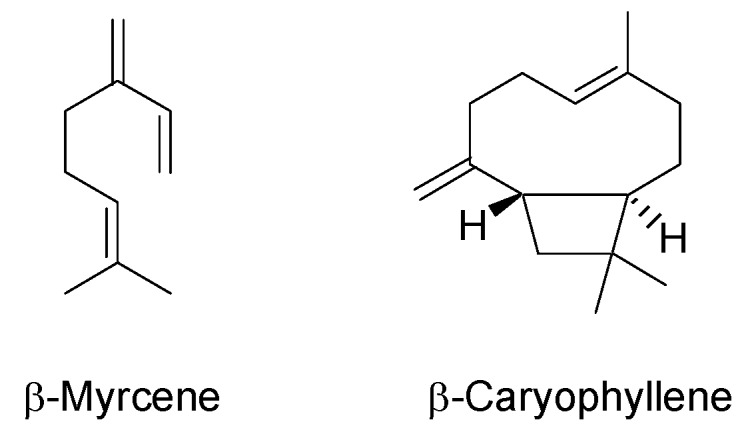
Chemical structures of main mono-and sesquiterpenes in hemp inflorescences.

Besides cannabinoids, volatile terpenes present in hemp essential oil (EO) are another relevant chemical class of compounds [5,14,15]. Indeed, more than 120 terpenes have been identified in *Cannabis*, contributing to its characteristic smell [5,15]. Hemp EO, in analogy with cannabinoids, is secreted by glandular trichomes present in the leaves and inflorescences of the plant, with α-pinene, β-myrcene and α-terpinolene being the most abundant compounds among monoterpens, and β-caryophyllene and α-humulene the main sesquiterpenes [14,15,16,17,18,19,20,21]. β-Myrcene (Figure 2) is believed to be associated with the sedative effects related to *Cannabis* product consumption, while α-pinene leads to the improvement of learning and memory through its anti-acetylcholinesterase action [14,16]. Regarding β-caryophyllene (Figure 2) and caryophyllene oxide, they are known to possess both analgesic and anti-proliferative activities [14,16]. In addition, hemp terpenes may enhance some biological properties ascribed to cannabinoids, due to their ability to increase blood-brain barrier permeability and to interact with neurotransmitter receptors [5,22,23]. 

The interest in the EO from hemp has been growing recently, as more and more studies have been focusing on its characterization and the assessment of its biological activity, especially for its antimicrobial and insecticidal properties [19,20,24]. Niessen et al. have tested the antimicrobial capacity of the EO of three industrial hemp varieties on both Gram-positive and Gram-negative bacteria, as well as on yeasts related to human commensals and phytopathogens, concluding that hemp EO can significantly inhibit microbial growth, though the number of samples used in that study was quite limited [20]. The efficacy of the EO from the variety Futura 75 has also been evaluated as an antimicrobial agent on bacterial strains isolated from a clinical environment, showing promising results [19]. Finally, Marini et al. have demonstrated that hemp EO may represent a useful agent to reduce the virulence of the food contaminant *Listeria monocytogenes*, with a possible application in the food processing industry [24]. In general, the limited number of EO samples tested, their non-exhaustive chemical characterization and the absence of a correlation between the composition and the biological activity, are the main drawbacks of the existing scientific research on the antibacterial activity of hemp EO [19,20,24]. To do this, an extensive chemical characterization of hemp EO by means of gas-chromatography (GC) techniques is pivotal. In addition, a screening of hemp EO from different varieties is also necessary to identify the samples with the most promising biological value. 

In the light of all the above, the present study was aimed at the extensive phytochemical characterization of 17 hemp EO belonging to different varieties, together with the evaluation of their antibacterial activity against some pathogenic and spoilage microorganisms isolated from food and food processing environments. The qualitative and semi-quantitative profile of the EO considered in this study was determined by means of GC-MS and GC-FID, respectively. In addition, a GC-MS method was developed and applied for the first time to quantify CBD in the EO in order to define its role in relation with the observed antibacterial activity. The inhibitory effects of hemp EO were evaluated by both the agar well diffusion assay and the minimum inhibitory concentration (MIC) determination. The MIC of the main terpenic compounds found in the EOs analysed and of CBD against the same bacteria was also determined, in comparison to those of common antibiotics.

## 2. Results and Discussion

### 2.1. Qualitative and Semi-Quantitative Analysis of Hemp EOs

**Figure 3:**
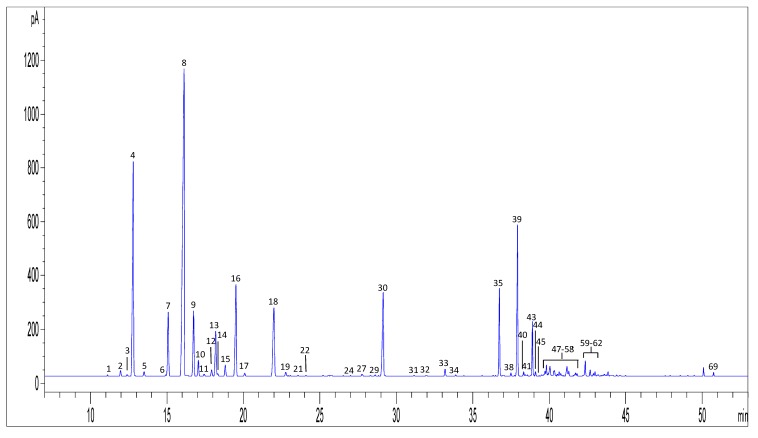
GC-FID chromatogram of a representative hemp EO (EO17). For peak numbering see Table 2.

Volatile compounds in hemp EOs were analysed by GC-FID and GC-MS and they were identified according to both their calculated linear retention indexes (LRI) and MS data, which were compared with available data in the literature and MS spectral libraries, respectively. Figure 3 shows a typical GC-FID chromatogram for a representative hemp EO (EO17). As shown in Table 1 and Table 2, the GC analysis allowed us to identify 71 compounds in hemp EOs, belonging to mono- and sesquiterpenes and cannabinoids. All the hemp EOs analysed in this study displayed almost the same qualitative and semi-quantitative profile: Monoterpenes were the most represented class of volatile compounds, ranging between 31% and 83% of the total peak area. In samples EO8 an EO9, the content of mono- and sesquiterpenes was balanced, while in EO1 and EO6 sesquiterpenes were more represented than monoterpenes, suggesting a possible aging. 

β-Myrcene (4.5–39.2%), α-pinene (4.8–25.4%), α-terpinolene (1.9–9.6%), β-pinene (3.4–8.2%), *trans*-ocimene (2.2–7.1%) and limonene (0.1–5.7%) are the most abundant compounds among monoterpenes. It is worth notice that the GC analysis of sample EO8 highlighted the presence of a particularly high percentage of linalool (3.7%) and terpinen-4-ol (1.1%), which was not observed for all the other samples. Samples EO3, EO12, EO13 and EO17 were the only ones which showed the presence of methyl chavicol (estragole) (1.8–6.2%) in their composition. EO3 and EO17 were also rich in geranyl acetate (1.6% and 4.3%, respectively).

As for the sesquiterpenes, β-caryophyllene was the most abundant compound in all the samples analysed, followed by α-humulene. Curiously, samples EO1, EO4, EO6, EO8, EO9, EO10 and EO14 had a particularly high content of caryophyllene oxide, reaching up to 9.5% in the last one. It should be pointed out that this compound represents an oxidation product of β-caryophyllene; therefore, its presence in the EO may be an indicator of either the low quality of the starting plant material from which the EO has been extracted or of non-optimal storage conditions or of the aging of the product. 

The GC data of the hemp EOs analysed in this work are in accordance with those previously described in the literature [5,14,15,16,17,18,19,20,21,25,26,27,28,29]. 

### 2.2. Analysis of Cannabinoids in Hemp EOs

**Figure 4:**
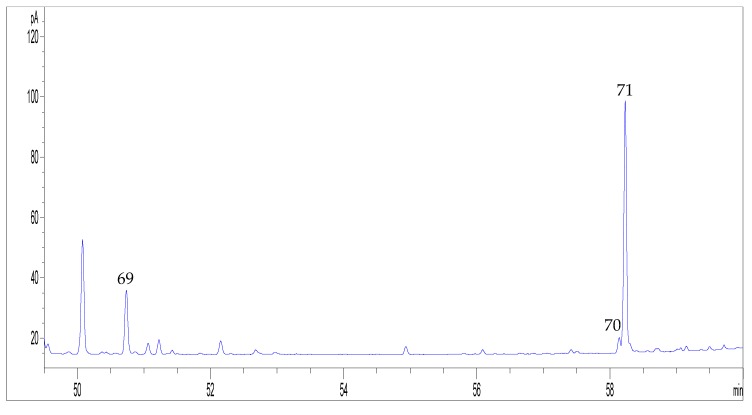
GC-FID chromatogram of a representative hemp EO (EO9), focused on the retention window of cannabinoids. For peak numbering, see Table 2.

It is worth notice that in the GC-FID chromatograms of the 17 EOs analysed in this work, the presence of cannabinoids was also observed (Figure 4). In particular, CBDV, CBC and CBD were found, though their percentage area was below 0.05% in most of the samples analysed.

For this reason, hemp EOs were further submitted to a quantitative analysis by GC-MS in order to quantify CBD, which represents the most abundant cannabinoid and also the most interesting one from the antimicrobial point of view. The analysis revealed a heterogeneous scenario in terms of CBD content among the samples. The vast majority of EOs displayed a CBD content in the range 0.1–1.0 mg/mL. Samples EO2, EO12, EO13, EO16 and EO17 were those with the lowest CBD amount, i.e., lower than 0.1 mg/mL. Conversely, samples EO6 and EO9 revealed a high content of this compound, corresponding to 1.7 mg/mL and 2.9 mg/mL, respectively.

### 2.3. Preliminary Screening by Means of the Agar Well Disk Diffusion Assay

A preliminary screening on the capacity of hemp EOs to inhibit bacterial growth on both Gram-positive and Gram-negative was performed by means of the agar well disk diffusion assay. The common antibacterial drugs ampicillin and ciprofloxacin were used as the positive controls. No antibiotic activity was displayed by the 17 hemp EOs on Gram-negative bacteria.

In relation to Gram-positive bacteria, in general, all the 17 EOs exhibited a good antimicrobial activity, expressed as an inhibition of the bacterial growth (Table S1). In particular, among the samples analysed, EO1, EO3, EO9, EO11, EO16 and EO17 showed good inhibition against *Enterococcus*, *Listeria* and *Staphylococcus* growth if compared to conventional antibiotics. Sample EO12 showed a very low activity towards all the microorganisms considered in this study and, therefore, it was not considered for further experiments.

### 2.4. Minimum Inhibitory Concentration (MIC)

The antimicrobial activity of the six EOs mentioned before (i.e., EO1, EO3, EO9, EO11, EO16 and EO17) was further confirmed by the determination of their MIC values on Gram-positive bacteria (Table 3).

The majority of hemp EOs did not exploit any antibacterial activity on *Staphylococcus* bacterial strains. Indeed, only three hemp EOs, including EO1, EO9, and EO11, exhibited a good activity against *Staphylococcus* strains, with an MIC lower than those determined by other authors [19].

With regard to *Listeria* and bacilli, the MIC values of these EOs were found to be similar to those of ampicillin. The anti-*Listeria* activity of the EO from hemp variety Futura has also been previously observed by Marini et al. [24].

Concerning *Enterococcus,* the situation is even more promising. As a matter of fact, these six EOs exhibited MIC values lower than those obtained with ciprofloxacin as the antibiotic agent. A good antibacterial activity was displayed by EO1, EO3, EO9 and EO11 against *Enterococcus*. In fact, the MIC values of these EOs were found to be lower than those of the antibiotic ciprofloxacin for some strains. Indeed, EO1 showed lower MIC values than ciprofloxacin for almost all the *Enterococcus* strains tested. EO3 was effective basically on two strains of *Enterococcus*, with MIC values of 8 to 10 times lower than those of ciprofloxacin. EO9 and EO11 showed good antibacterial activity on the majority of *Enterococcus* strains, with MIC values lower or equal to those of common antibacterial agents. Other authors have already described the antimicrobial activity of hemp EOs against *Enterococcus*, with particular regard to the Carmagnola and Futura varieties [20]. Indeed, Nissen et al. have observed a better activity of the Futura EO against *Enterococcus* with respect to the Carmagnola EO [20]. Nevertheless, our results showed a wider spectrum of action for the Futura EO (EO9) compared to the Carmagnola EO (EO3), but, for the latter, lower MIC values were found against the same bacterial strains with respect to those of the Futura one. In addition, Niessen et al. have demonstrated that the Futura EO was able to inhibit both Gram-positive and Gram-negative bacteria [20], while no antibacterial activity was exerted by the 17 hemp EOs considered in this study against Gram-negative bacteria.

As for bacilli, sample EO1 and EO3 showed better antibacterial activity, with MIC values comparable to those of ampicillin. In particular, EO1 and EO3 showed an MIC value lower than that of ampicillin against *Bacillus cereus*.

The antibiotic activity of pure CBD and of the major terpenes detected in hemp EOs was also evaluated in the present study. In particular, the MIC values of pure α-pinene, β-pinene, β-myrcene, α-terpinolene, β-caryophyllene and CBD were calculated. The results are shown in Table 4. In general, all the pure compounds tested in this work exhibited an antibacterial activity towards the strains considered. In particular, a good antibiotic activity was observed for CBD and for the monoterpenes (α-pinene, β-pinene and β-myrcene), especially toward *Listeria* and *Enterococcus* strains, in accordance with Nissen et al. [20]. Concerning *Staphylococcus* and *Bacillus* strains, all the tested compounds exhibited MIC values higher than those of common antiobiotics, with the exception of α-pinene and β-pinene, which showed lower MIC values than amoxicillin against *B. cereus*. 

### 2.5. Chemical Composition–Bioactivity Relationships

By combining the MIC values obtained on bacterial strains of the 17 hemp EOs with their chemical composition, it is difficult to identify the compounds responsible for the antimicrobial activity. The EOs that exhibited the higher antibacterial activity were EO1, EO3, EO9, EO11, EO13, EO16 and EO17. Among the pure compounds tested on the bacterial strains, CBD, α-pinene, β-pinene and β-myrcene displayed the best antibacterial activity; therefore, their content in the EOs was taken into account. To simplify the discussion, the chemical composition–bioactivity relationships will be discussed separately for each bacterial species.

Regarding *Staphylococcus* strains, EO1, EO9 and EO11 displayed the best activity toward these pathogenic agents. All of them showed a discrete or high (EO9) amount of CBD, while they had low relative percentages of the above cited monoterpenes, with the only exception of EO11, which showed good relative percentaces of α- and β-pinene. So, the antibacterial activity of these EOs may be due to the content of CBD. Nevertheless, pure CBD did not show a remarkable activity toward *Staphylococcus* strains. Therefore, other terpenic compounds in the above mentioned EOs may exert a synergistic effect with CBD, maybe by enhancing its penetration into bacterial cells.

In the case of *Listeria*, all the isolated compounds showed good MIC values, especially α- and β-pinene. However, the hemp EOs exhibiting good anti-*Listeria* activity had a very different relative percentage of these two compounds, making a composition–activity relationship difficult.

Concerning *Enterococcus* strains, CBD, α-pinene, β-pinene and β-myrcene exhibited a higher antibacterial activity in comparison with ciprofloxacin. By looking at the chemical composition of the EOs active on *Enterococcus*, it is possible to notice that they are all characterized by a discrete or quite high amount of CBD, but the relative percentage of monoterpenes differs a lot among them. As a matter of fact, EO1 and EO9 are those with the lowest amount of α-pinene, β-pinene and β-myrcene, while EO3 and EO11 had a high relative percentage of monoterpenes.

Finally, EO1 and EO3 were active against *Bacillus* strains, in particular against *B. cereus*. Both α- and β-pinene exhibited a better antibiotic activity than amoxicillin on this species, but by looking at the chemical composition, it is clear that EO1 and EO3 were not those with the highest relative percentage of these two compounds.

The antibacterial activity of hemp EOs may probably arise from a synergism between the different compounds present in this rich phytocomplex.

## 3. Materials and Methods

### 3.1. Chemicals and Solvents

CBD standard solution (1 mg/mL in methanol) was purchased from Cerilliant (Round Rock, TX, USA). α-Pinene, β-pinene, β-myrcene, α-terpinolene and β-caryophyllene were purchased from Extrasynthese (Genay, France). *n*-Hexane GC-grade was purchased from VWR (Milan, Italy).

### 3.2. Hemp EO Samples

The samples from EO2 to EO5, EO7, EO8 and from EO10 to EO17 were kindly provided by Hemp Service International (France). Samples EO1, EO6 and EO9 were gifted by Prof. Steven Bazzani of the I.I.S L. Spallanzani (Montombraro, Modena, Italy). All hemp EOs were obtained by steam distillation of the inflorescences or the whole plants, which were certified for a content of Δ^9^-THC below 0.2% (*w*/*w*). All the samples considered in this study were approved for commercial use by the European Union [30]. In particular, the following hemp varieties were considered in this study: Antàl (EO1), Bielobrzerski (EO2), Carmagnola (EO3), Carmagnola CS (EO4), Dioica 88 (EO5), Fedora 17 (EO6), Ferimon (EO7), Finola (EO8), Futura 75(EO9), KC Virtus (EO10), KC Zuzana (EO11), Markant (EO12), Santhica 27 (EO13), Santhica 70 (EO14), Tiborszallasi (EO15), Tygra (EO1) and Zenith (EO17).

### 3.3. GC-MS Analysis of Volatile Compounds

Analyses were performed on a 7820A gas chromatograph coupled with a 5975C network mass spectrometer (GC-MS) (Agilent Technologies, Waldbronn, Germany). Compounds were separated on an Agilent Technologies HP-5 MS cross-linked poly-5% diphenyl–95% dimethyl polysiloxane (30 m × 0.32 mm inner diameter (i.d.), 0.25 μm film thickness) capillary column. The column temperature was initially set at 45 °C, then increased at a rate of 2 °C/min up to 100 °C, then raised to 250 °C at a rate of 5 °C/min, and again raised up to 280 °C at a rate of 11 °C/min, and finally held for 15 min. The injection volume was 0.1 μL, with a split ratio of 1:40. Helium was used as the carrier gas, at a flow rate of 0.7 mL/min. The injector, transfer line and ion-source temperatures were 250, 280 and 230 °C, respectively. MS detection was performed with electron ionization (EI) at 70 eV, operating in the full-scan acquisition mode in the *m*/*z* range 40–400. The EOs were diluted 1:20 (*v*/*v*) with *n*-hexane before GC-MS analysis. All reference standards used for GC analysis, chromatographic grade organic solvents and reagents were purchased from Sigma-Aldrich (Milan, Italy).

### 3.4. GC-FID Analysis of Volatile Compounds

Analyses were carried out on a 7820A GC coupled with a flame ionization detector (FID) from Agilent Technologies. Compounds were separated on an Agilent Technologies HP-5 cross-linked poly-5% diphenyl–95% dimethyl polysiloxane (30 m × 0.32 mm i.d., 0.25 mm film thickness) capillary column. The temperature program was the same as described above. The injection volume was 0.1 μL in the split mode 1:20. Helium was used as the carrier gas at a flow rate of 1.0 mL/min. The injector and detector temperature were set at 250 and 300 °C, respectively. The EOs and the reference standards were diluted to 1:20 (*v*/*v*) with *n*-hexane before GC-FID analysis. The analyses were performed in triplicate for each sample.

### 3.5. Qualitative and Semi-Quantitative Analysis of Volatile Compounds

The compounds in the EOs analysed were identified by comparing the retention times of the chromatographic peaks with those of authentic reference standards run under the same conditions and by comparing the experimental LRI values, calculated from a mixture of *n*-alkanes (C_8_–C_40_) in *n*-hexane and injected under the same conditions as those previously described in the literature. Peak enrichment by co-injection with authentic reference compounds was also carried out. Comparison of the MS-fragmentation pattern of the target analytes with those of pure components was performed. A mass-spectrum database search was performed by using the National Institute of Standards and Technology (NIST, Gaithersburg, MD, USA) mass-spectral database (version 1.4). The percentage relative amount of individual components was expressed as percent peak area relative to total peak area.

### 3.6. Quantitative GC-MS Analysis of Cannabinoids

All quantitative GC-MS analyses of cannabinoids were performed on a Saturn 2200 ion trap-quadrupole system (Varian, Walnut Creek, CA, USA), equipped with a DB-5MS capillary column (30m × 0.25mm i.d., 0.25 μm film thickness, Agilent, SantaClara, CA, USA). The column temperature was initially set at 150 °C, held for 1 min and then increased at a rate of 10 °C/min up to 230 °C, which was held for 1 min. Then, the temperature was raised to 270 °C at a rate of 3 °C/min and finally increased at a rate of 10 °C/min up to 290 °C and held for 3 min. The injection volume was 1 μL, with a split ratio of 1:30. Helium was used as the carrier gas, at a flow rate of 1.0 mL/min. The injector, transfer line and source temperature were set at 250, 290 and 250 °C, respectively. MS detection was performed with electron ionization (EI) at 70 eV, operating in the full-scan acquisition mode in the *m*/*z* range 43–510. The EOs were diluted to 1:10 (*v*/*v*) with MeOH before GC-MS analysis. For CBD calibration curve, the concentration range was 5–100 ng/μL. (*r*^2^ = 0.994). For the quantification of CBD, the transition 314.3→231.4 *m*/*z* was monitored.

### 3.7. Bacterial Strains

In this study, classified bacteria (ATCC—American Type Culture Collection, and NCTC—National Collection of Type Cultures) and some pathogenic and spoilage microorganisms isolated from food and food environments were used as indicators. Tryptic Soy Agar (TSA, Oxoid) and Tryptic Soy Broth (TSB, Oxoid) were used as the growth media.

### 3.8. Agar Well Disk Diffusion Assay

Seventeen hemp EOs were tested for their antibacterial activity by the agar disk diffusion assay, according to the standard procedure of the Clinical and Laboratory Standards Institute [31] with slight modifications. Plates containing Tryptic Soy Agar, a non-selective culture medium, were uniformly spread with 100 μL of 10^6^ CFU/mL of each strain suspensions. Then, sterile disks of 6 mm in diameter, containing 10 μL of each EO, were placed on the agar surfaces. Ampicillin (2 μg) and ciprofloxacin (5 μg) discs were used as the positive control for *Listeria* and *Bacillus*, and *Enterococcus* and *Staphylococcus*, respectively. After incubation at 37 °C for 24 h, a clear zone of inhibition of the bacterial growth, expressed in millimeters (mm), was measured to quantify the EO antibacterial activity [32].

### 3.9. Minimum Inhibitory Concentration (MIC) Assay

A microwell dilution method was used to determine the minimum inhibitory concentration (MIC) values of all varieties of hemp EOs against each tested bacterium, following the guidelines of the Clinical and Laboratory Standards Institute (CLSI 2012) [31]. The assay was performed in sterile 96-well microplates by dispensing into each well 95 µL of nutrient broth and 5 µL of bacterial suspensions, to final inoculums concentrations of 106 CFU/mL. Then, 100 µL of EO serial dilutions were added to obtain concentrations ranging from 512 to 0.25 μL EO/mL [33]. The last well, containing 195 μL of nutrient broth and 5 μL of bacterial strain without EO, was used as a negative control. The antibiotics ampicillin and ciprofloxacin diluted in the nutrient broth with strains added were used as the positive control. The plates were incubated at 37 °C for 24 h. The MIC was defined as the lowest concentration of the EO that inhibited visible growth of the tested microorganisms after the optical density (OD) measured at 570 nm, using a microtiter plate reader. The MIC values was expressed as μg/mL, by taking into account the density value for each EO. All the experiments were repeated three times.

## 4. Conclusions

In the present study, a deep characterisation of 17 EOs from different hemp varieties, by means of GC-FID and GC-MS analysis, was carried out. In total, 71 compounds were identified in the GC chromatograms of the samples, belonging to monoterpenes, sesquiterpenes and cannabinoids. The amount of CBD in the hemp EOs was also determined by GC-MS. The antimicrobial activity of these EOs toward both Gram-positive and Gram-negative bacteria isolated from food and food environments was assessed. As a comparison, the MIC values of pure CBD and of the most representative terpenes in the EOs were determined.

Overall, the results obtained in this study demonstrate that hemp EO represents a promising product against Gram-positive bacteria, while it proved to be ineffective towards the Gram-negative ones. The chemical composition–bioactivity relationships did not show a clear correlation between the relative amount of the most representative compounds in the EOs and their bioactivity, thus leading to the possible conclusion that the antibacterial activity displayed by hemp EOs is more likely to be due to synergistic interactions among different compounds.

In conclusion, the results obtained in this study demonstrate that hemp EO can inhibit or reduce bacterial proliferation, thus proving to be a valid support to reduce microorganism contamination, especially in the food processing field. Further experiments should be performed in order to understand the main components of the EOs responsible for its antibacterial activity by assessing their synergism.

## Figures and Tables

**Table 1:** Qualitative and semi-quantitative analysis of hemp essential oils (EOs) analysed in this study. Data are expressed as mean (*n* = 3) percentage peak area ± SD.

Peak n.	Compound	LRI	LRI lit ^b^	EO1	EO2	EO3	EO4	EO5	EO6	EO7	EO8
1	Heptanal	903	901	-	-	0.2 ^a^	-	-	-	0.3 ^a^	-
2	5,5-Dimethyl-1-vinylbicyclo[2.1.1]-hexane	918	921	0.1 ^a^	-	0.5 ^a^	0.5 ^a^	0.4 ^a^	0.3 ^a^	0.4 ^a^	0.4 ^a^
3	α-Thujene	926	924	-	-	-	0.2 ^a^	0.1 ^a^	0.1 ^a^	0.1 ^a^	-
4	α-Pinene	934	931	7.4 ± 0.3	19.4 ± 0.1	14.6 ± 0.4	18.6 ± 0.2	20.4 ± 0.2	4.8 ± 0.1	16.0 ± 0.1	11.0 ± 0.1
5	Camphene	947	947	0.2 ^a^	-	0.3 ^a^	0.4 ^a^	0.4 ^a^	0.1 ^a^	0.3 ^a^	0.4 ^a^
6	β-Thujene	973	966	0.1 ^a^	-	0.1 ^a^	-	0.1 ^a^	0.1 ^a^		-
7	β-Pinene	976	978	3.4 ± 0.1	7.8 ^a^	5.5 ± 0.1	6.1 ± 0.2	8.2 ± 0.1	2.1 ± 0.1	6.5 ^a^	4.1 ^a^
8	β-Myrcene	994	992	8.3 ^a^	29.5 ± 0.3	34.4 ± 1.3	25.9 ± 1.3	30.4 ± 0.5	12.5 ± 0.8	33.5 ± 0.3	4.5 ± 0.1
9	α-Phellandrene	1006	1007	0.1 ^a^	-	1.6 ± 0.1	0.4 ± 0.2	0.3 ^a^	0.2 ^a^	0.3 ^a^	-
10	Δ^3^-Carene	1010	1010	0.2 ^a^	-	0.6 ^a^	0.8 ^a^	0.7 ^a^	0.9 ± 0.1	0.5 ^a^	-
11	α-Terpinene	1016	1017	0.1 ^a^	-	0.3 ^a^	0.2 ^a^	0.3 ^a^	0.2 ^a^	0.3 ^a^	0.3 ^a^
12	p-Cymene	1024	1026	-	-	0.2 ^a^	0.2 ^a^	0.1 ^a^	0.3 ^a^		-
13	Limonene	1028	1035	0.1 ^a^	5.7 ± 0.2	4.3 ± 0.2	4.2 ± 0.4	5.5 ^a^	1.2 ± 0.1	4.2 ^a^	1.9 ^a^
14	1,8-Cineole	1030	1032	1.8 ^a^	-	0.3 ^a^	0.9 ± 0.1 ^a^	0.2 ^a^	0.1 ^a^	0.2 ^a^	1.8 ^a^
15	*cis*-Ocimene	1038	1040	0.3 ^a^	-	0.4 ^a^	0.6 ^a^	0.5 ^a^	0.8 ^a^	0.5 ^a^	0.5 ^a^
16	*trans*-Ocimene	1049	1050	5.0 ± 0.1	4.9 ± 0.1	4.2 ± 0.2	4.2 ± 0.1	4.0 ± 0.1	5.0 ^a^	4.6 ^a^	2.2 ^a^
17	γ-Terpinene	1058	1062	0.1 ^a^	-	0.3 ^a^	0.3 ^a^	0.2 ^a^	0.2 ^a^	0.3 ^a^	0.2 ^a^
18	α-Terpinolene	1089	1088	3.4 ± 0.1	7.5 ± 0.1	8.3 ± 0.5	5.4 ± 0.3	8.2 ± 0.2	6.6 ± 0.2	8.4 ± 0.1	1.9 ± 0.1
19	Linalool	1101	1104	-	-	0.4 ^a^	0.4 ^a^	0.2 ^a^	0.1 ^a^	0.3 ^a^	3.7 ^a^
20	6-Camphenol	1105	1110	0.1 ^a^	-	-	0.1 ^a^	-	-	0.1 ^a^	-
21	Fenchol	1113	1114	0.1 ^a^	-	0.3 ± 0.1	0.2 ^a^	0.2 ^a^	-	0.2 ^a^	-
22	neo-Alloocimene	1121	1143	-	-	-	0.1 ^a^	0.1 ^a^	-	0.2 ^a^	-
23	*trans*-Pinocarveol	1138	1139	-	-	-	0.1 ^a^	-	-	0.1 ^a^	0.2 ^a^
24	Camphor	1144	1145	-	-	-	-	-	-	0.2 ^a^	0.9 ^a^
25	Isopulegol	1146	1156	-	-	-	-	-		-	-
26	Borneol	1166	1166	-	-	-	-	-	-	0.1 ^a^	0.4 ^a^
27	Terpinen-4-ol	1178	1177	-	-	0.6 ^a^	0.2 ^a^	0.1 ^a^	0.1 ^a^	0.2 ^a^	1.1 ^a^
28	p-Cymen-8-ol	1190	1183	-	-	-	-	-	0.2 ^a^	-	-
29	α-Terpineol	1191	1193	-	-	0.2 ^a^	0.1 ^a^	0.1 ^a^	-	0.2 ^a^	0.5 ± 0.1
30	Methyl chavicol	1199	1196	0.1 ^a^	-	1.8 ^a^	-	-	0.1 ^a^	-	-
31	Neral	1243	1249	-	-	-	-	-	-	-	-
32	Linalyl acetate	1261	1253	0.1 ^a^	-	-	0.1 ^a^	-		-	4.3 ^a^
33	Bornyl acetate	1289	1287	-	-	-	-	-	-	-	-
34	Thymol	1301	1297	0.1 ^a^	-	-	0.1 ^a^		0.1 ^a^		0.4 ^a^
35	Geranyl acetate	1388	1392	-	-	1.6 ± 0.1	0.5 ^a^	0.1 ^a^	0.3 ^a^	0.1 ^a^	0.3 ± 0.1
36	β-Bourbonene	1389	1386	0.1 ^a^	-	-	0.1 ^a^	-	0.1 ^a^	-	-
37	α-Gurjunene	1411	1408	0.3 ^a^	-	-	0.3 ^a^	0.2 ^a^	0.6 ^a^	0.2 ^a^	0.8 ^a^
38	Isocaryophyllene	1417	1411	0.2 ^a^	-	0.2 ^a^	-	-	-	-	0.4 ^a^
39	β-Caryophyllene	1427	1428	21.6 ± 0.2	20.0 ± 0.6	8.5 ± 0.3	14.3 ± 0.5	9.3 ± 0.2	21.1 ± 0.3	10.3 ± 0.4	29.8 ± 0.2
40	α-Bergamotene	1441	1432	1.6 ± 0.1	-	0.2 ^a^	0.3 ^a^	0.2 ^a^	2.4 ± 0.1	0.3 ^a^	1.9 ^a^
41	Aromadendrene	1445	1449	0.1 ^a^	-	-	0.2 ^a^	0.1 ^a^	0.3 ^a^	0.1 ^a^	0.9 ^a^
42	Aristolene	1453	1450	-	-	-	0.1 ^a^	-	-	-	0.3 ^a^
43	α-Humulene	1461	1455	10.1 ± 0.1	5.3 ± 0.3	2.8 ± 0.1	4.6 ± 0.2	2.9 ± 0.1	9.7 ± 0.2	3.3 ± 0.1	10.1
44	Alloaromadendrene	1468	1467	1.8 ± 0.1	-	0.3 ^a^	0.6 ± 0.1	0.2 ^a^	1.0 ± 0.2	0.3 ^a^	0.7 ^a^
45	γ-Muurolene	1482	1477	0.3 ^a^	-	0.1 ^a^	0.2 ^a^	-	0.5 ^a^	0.1 ^a^	0.9 ^a^
46	Germacrene D	1487	1485	0.2 ^a^	-	-	0.1 ^a^	-	0.3 ^a^	0.1 ^a^	-
47	β-Selinene	1490	1489	0.2 ^a^	-	0.2 ^a^	0.4 ^a^	0.2 ^a^	-	0.3 ^a^	2.9 ± 0.6
48	α-Selinene	1493	1494	1.0 ± 0.3	-	0.5 ^a^	0.9 ± 0.1	0.5 ^a^	2.1 ± 0.2	0.6 ^a^	-
49	Valencene	1497	1496	3.0 ± 0.4	-	0.1 ^a^	0.1 ^a^	0.1 ^a^	0.1 ^a^	0.1 ^a^	-
50	β-Bisabolene	1501	1505	2.7 ± 0.4	-	0.6 ± 0.1	0.9 ^a^	0.5 ^a^	1.8 ± 0.1	0.6 ^a^	2.2 ± 0.2
51	α-Muurolene	1512	1497	0.7 ± 0.2	-	0.3 ± 0.1	0.6	0.4	0.7 ± 0.1	0.4	1.6 ± 0.1
52	α-Patchoulene	1521	1445	0.2 ^a^	-	0.1 ^a^	0.1 ^a^	-	0.3 ^a^	0.3 ^a^	0.4 ^a^
53	γ-Cadinene	1525	1512	0.5 ± 0.1	-	0.3 ^a^	0.2 ^a^	0.2 ^a^	0.3 ^a^	0.2 ^a^	-
54	δ-Cadinene	1529	1519	0.5 ± 0.2	-	0.2 ^a^	0.2 ^a^	0.2 ^a^	0.7 ± 0.1	-	0.8 ^a^
55	δ-Selinene	1545	1540	1.1 ± 0.3	-	0.7 ± 0.1	0.7 ± 0.1	0.8 ^a^	1.3 ^a^	0.9 ^a^	0.4 ± 0.1
56	Selina-3,7(11)-diene	1550	1545	0.4 ^a^	-	0.3 ± 0.1	0.3 ^a^	0.2 ^a^	1.3 ± 0.2	0.3 ^a^	-
57	*trans*-Nerolidol	1568	1562	1.0 ± 0.2	-	-	0.2 ^a^	0.2 ^a^	0.7 ^a^	0.2 ^a^	0.2 ^a^
58	Spathulenol	1585	1577	0.1 ^a^	-	-	-	-	0.2 ^a^	-	-
59	Caryophyllene oxide	1593	1583	9.5 ± 0.5	-	0.8 ± 0.1	1.3 ± 0.1	0.8 ± 0.1	6.6 ± 0.2	0.8	2.5 ± 0.1
60	Viridiflorol	1607	1600	0.4 ^a^	-	0.1 ^a^	-	-	0.3 ^a^	-	-
61	Guaiol	1619	1602	2.6 ± 0.3	-	-	0.3 ^a^	0.2 ^a^	1.8 ± 0.1	0.2 ^a^	0.5 ^a^
62	Humulene-1,2-epoxide	1621	1615	-	-	0.2 ^a^	-	-	-	-	-
63	Caryophylla-4(12),8(13)-dien-5-ol	1628	1634	0.5 ^a^	-	-	-	0.2 ^a^	0.3 ^a^	0.2 ^a^	-
64	epi-γ-Eudesmol	1636	1625	0.2 ^a^	-	0.1 ^a^	-	0.1 ^a^	0.2 ^a^	0.1 ^a^	-
65	Cubenol	1643	1643	-	-	0.1 ^a^	-	0.1 ^a^	-	0.1 ^a^	-
66	(*Z*)-14-Hydroxycaryophyllene	1667	1666	0.2 ^a^	-	-	-	-	0.5 ^a^	-	-
67	epi-α-Bisabolol	1681	1683	0.8 ± 0.2	-	0.1 ^a^	-	-	0.7 ± 0.1	-	-
68	β-Bisabolol	2041	-	0.1 ^a^	-	-	-	-	0.2 ^a^	-	-
69	Cannabidivarin (CBDV)	2193	2208	-	-	0.2 ^a^	0.1 ^a^	0.2 ^a^	0.1 ^a^	0.2 ^a^	-
70	Cannabichromene (CBC)	2772	-	-	-	-	-	-	-	-	-
71	Cannabidiol (CBD)	2779	-	0.1 ^a^	-	-	-	-	0.3 ^a^	-	0.1 ^a^
	Total area			92.9 ± 1.4	100.0	97.5 ± 1.4	98.1 ± 0.7	98.7 ± 0.2	92.1 ± 0.8	98.0 ± 0.5	98.4 ± 1.0

Experimental conditions as in Section 3.3 and Section 3.4. Percentage peak areas below 0.05% are indicated with -. ^a^ SD < 0.05. ^b^ LRI data from the NIST Chemistry WebBook, SRD 69, https://www.nist.gov.

**Table 2:** Qualitative and semi-quantitative analysis of hemp EOs analysed in this study. Data are expressed as mean (*n* = 3) percentage peak area ± SD.

Peak n.	Compound	LRI	LRI lit ^b^	EO9	EO10	EO11	EO12	EO13	EO14	EO15	EO16	EO17
1	Heptanal	903	901	-	-	-	0.3 ^a^	0.1 ^a^	0.3 ^a^	0.1 ^a^	0.1 ^a^	-
2	5,5-Dimethyl-1-vinylbicyclo[2.1.1]-hexane	918	921	0.3 ^a^	0.5 ^a^	-	0.3 ^a^	0.4 ^a^	1.4 ^a^	0.3 ± 0.1	0.4 ^a^	0.3 ^a^
3	α-Thujene	926	924	0.1 ^a^	0.1 ^a^	-	0.2 ^a^	0.1 ^a^	0.1 ^a^	0.1 ^a^	0.1 ^a^	0.1 ^a^
4	α-Pinene	934	931	8.4 ± 0.3	25.4 ± 0.3	20.7 ± 1.6	14.6 ± 0.4	15.5 ± 0.1	11.1	16.6 ± 0.3	20.3 ± 0.5	14.7 ± 0.4
5	Camphene	947	947	0.2 ^a^	0.5 ^a^	0.4 ± 0.1	0.3 ^a^	0.3 ^a^	0.2 ^a^	0.3 ^a^	0.4 ^a^	0.3 ± 0.1
6	β-Thujene	973	966	0.1 ^a^	-	-	0.1 ^a^	0.1 ^a^	-	-	-	-
7	β-Pinene	976	978	3.2 ± 0.1	9.2 ± 0.1	6.9 ± 0.5	4.2 ± 0.1	5.3 ± 0.1	5.1 ± 0.1	5.3 ± 0.1	8.0 ± 0.1	4.1 ± 0.1
8	β-Myrcene	994	992	19.8 ± 0.5	19.8 ± 0.2	14.5 ± 1.0	29.0 ± 0.4	33.4 ± 0.1	29.9 ± 0.2	39.2 ± 2.1	33.4 ± 0.5	28.9 ± 2.4
9	α-Phellandrene	1006	1007	0.3 ^a^	0.2 ^a^	0.3 ^a^	3.8 ± 0.1	2.7 ^a^	0.2 ^a^	0.3 ^a^	0.3 ^a^	3.9 ± 0.3
10	Δ^3^-Carene	1010	1010	0.7 ^a^	0.7 ^a^	0.6 ^a^	1.02 ^a^	0.9 ^a^	0.5 ^a^	1.1 ^a^	0.8 ^a^	1.0 ^a^
11	α-Terpinene	1016	1017	0.3 ^a^	0.2 ^a^	0.3 ^a^	0.1 ^a^	0.2 ^a^	0.2 ^a^	0.2 ^a^	0.3 ^a^	0.1 ^a^
12	p-Cymene	1024	1026	0.1 ^a^	0.1 ^a^	-	0.6 ^a^	0.2 ^a^	0.2 ^a^	-	0.1 ^a^	0.4 ^a^
13	Limonene	1028	1035	2.1 ^a^	5.3 ± 0.1	3.4 ± 0.2	3.0 ^a^	4.1 ^a^	2.1 ^a^	2.8 ± 0.1	5.6 ± 0.1	2.9 ± 0.2
14	1,8-Cineole	1030	1032	0.1 ^a^	0.7 ^a^	0.2 ^a^	0.2 ^a^	0.2 ^a^	0.3 ^a^	-	0.2 ^a^	0.2 ^a^
15	*cis*-Ocimene	1038	1040	0.6 ^a^	0.5 ^a^	0.6 ^a^	0.7 ^a^	0.6 ^a^	1.3 ^a^	0.5 ^a^	0.3 ^a^	0.7 ± 0.1
16	*trans*-Ocimene	1049	1050	4.9 ± 0.2	3.1 ^a^	6.3 ± 0.4	6.3 ± 0.1	5.6 ^a^	7.1 ± 0.1	6.4 ± 0.7	3.2 ± 0.1	6.2 ± 0.6
17	γ-Terpinene	1058	1062	0.2 ^a^	0.3 ^a^	0.3 ^a^	-	0.2 ^a^	0.3 ^a^	2.8 ± 0.6	0.2 ^a^	0.2 ^a^
18	α-Terpinolene	1089	1088	9.6 ± 0.4	4.0 ± 0.1	6.3 ± 0.3	6.4 ^a^	7.5 ^a^	3.5 ^a^	5.2 ^a^	7.4 ± 0.2	6.2 ± 0.3
19	Linalool	1101	1104	0.2 ^a^	0.6 ^a^	-	0.3 ^a^	0.4 ^a^	-	0.3 ^a^	0.3 ^a^	0.3 ± 0.1
20	6-Camphenol	1105	1110	0.1 ^a^	-	-	-	0.1 ^a^	0.6 ^a^	-	0.1 ^a^	-
21	Fenchol	1113	1114	-	0.1 ^a^	-	-	0.2 ^a^	-	0.1 ^a^	0.3 ± 0.1	0.1 ^a^
22	neo-Alloocimene	1121	1143	-	0.1 ^a^	-	-	0.1 ^a^	-	0.1 ^a^	0.2 ^a^	0.1 ^a^
23	*trans*-Pinocarveol	1138	1139	-	0.1 ^a^	-	-		-	-	0.1 ^a^	-
24	Camphor	1144	1145	0.1 ^a^	0.1 ^a^	-	0.1 ^a^		-	-	-	0.1 ^a^
25	Isopulegol	1146	1156		-	-	-		-	-	0.2 ^a^	-
26	Borneol	1166	1166	-	0.2 ^a^	-	-		-	-	0.1 ^a^	-
27	Terpinen-4-ol	1178	1177	0.1 ^a^	0.4 ^a^	0.3 ^a^	0.2 ^a^	0.3 ^a^	0.2 ^a^	0.1 ^a^	0.2 ^a^	0.2 ^a^
28	p-Cymen-8-ol	1190	1183	0.1 ^a^	-	-	-		-	-	0.2 ^a^	-
29	α-Terpineol	1191	1193	-	0.2 ^a^	-	-	0.2 ^a^	-	0.1 ^a^	0.1 ^a^	0.1 ^a^
30	Methyl chavicol	1199	1196	0.1 ^a^		-	6.2 ^a^	3.6 ± 0.1	-	0.1 ^a^	-	5.9 ± 0.6
31	Neral	1243	1249	-	1.4 ± 0.1	-	-		-	-	-	0.1 ^a^
32	Linalyl acetate	1261	1253	0.1 ^a^	0.1 ^a^	-	-		-	-	0.1 ^a^	0.1 ^a^
33	Bornyl acetate	1289	1287	-	-	-	0.4 ^a^		-	-	0.1 ^a^	0.4 ^a^
34	Thymol	1301	1297	-	0.3 ^a^	-	-	-			-	0.1 ^a^
35	Geranyl acetate	1388	1392	0.2 ^a^	0.1 ^a^	-	4.3 ± 0.3	0.5 ^a^	0.2 ^a^	0.1 ^a^	0.4 ^a^	4.3 ± 0.4
36	β-Bourbonene	1389	1386	0.1 ^a^	-	-	-	-	-	-	0.1 ^a^	-
37	α-Gurjunene	1411	1408	0.4 ^a^	0.4 ^a^	-	-	0.2 ^a^	0.5 ^a^	0.2 ± 0.1	-	-
38	Isocaryophyllene	1417	1411	-	-	0.4 ^a^	0.1 ^a^		-	-	0.2 ^a^	0.2 ^a^
39	β-Caryophyllene	1427	1428	14.8 ± 0.5	13.1 ± 0.4	22.3 ± 0.5	7.6 ± 0.6	8.2 ± 0.3	14.6 ± 0.3	9.2 ± 0.4	7.6 ± 0.5	7.7 ± 0.6
40	α-Bergamotene	1441	1432	1.4 ± 0.2	0.3 ^a^	1.3 ^a^	0.2 ^a^	0.2 ^a^	1.0 ^a^	0.2 ± 0.1	0.2 ^a^	0.2 ^a^
41	Aromadendrene	1445	1449	0.2 ^a^	0.2 ^a^	0.6 ± 0.1	0.1 ^a^	0.1 ^a^	-	0.1 ^a^	0.1 ^a^	0.1 ^a^
42	Aristolene	1453	1450	-	-	-	-		-	-	-	-
43	α-Humulene	1461	1455	6.6 ± 0.1	3.8 ± 0.2	7.5 ± 0.2	2.5 ± 0.3	2.5 ± 0.1	5.3 ± 0.1	2.7 ± 0.2	2.2 ± 0.2	2.5 ± 0.2
44	Alloaromadendrene	1468	1467	0.7 ^a^	0.5 ^a^	0.4 ^a^	0.2 ^a^	0.2 ^a^	0.9 ^a^	0.2 ± 0.1	0.2 ^a^	0.2 ^a^
45	γ-Muurolene	1482	1477	0.4 ^a^	0.1 ^a^	0.6 ^a^	-	0.1 ^a^	-	0.1 ^a^	0.1 ^a^	0.1 ^a^
46	Germacrene D	1487	1485	0.7 ^a^	0.1 ^a^	-	0.1 ^a^	0.1 ^a^	-	-	0.1 ^a^	-
47	β-Selinene	1490	1489	0.4 ^a^	0.3 ^a^	0.5 ^a^	0.5 ^a^	-	0.4 ^a^	0.2 ^a^	0.2 ^a^	0.3 ^a^
48	α-Selinene	1493	1494	1.3 ± 0.2	0.6 ^a^	1.5 ± 0.1	0.1 ^a^	0.5 ± 0.1	1.0 ^a^	0.4 ^a^	0.4 ^a^	0.5 ± 0.1
49	Valencene	1497	1496	0.2 ^a^	0.1 ^a^	-	-	0.1 ^a^	0.2 ^a^	0.1 ^a^	0.1 ^a^	0.1 ^a^
50	β-Bisabolene	1501	1505	1.3 ± 0.1	0.7 ^a^	1.5 ± 0.1	0.7 ^a^	0.5 ^a^	0.9 ^a^	0.5 ^a^	0.5 ^a^	0.7 ± 0.1
51	α-Muurolene	1512	1497	0.5 ± 0.1	0.5	1.3 ± 0.1	0.4	0.4	0.7	0.3 ± 0.1	0.2	0.5 ± 0.1
52	α-Patchoulene	1521	1445	0.2 ^a^	0.1 ^a^	-	0.1 ^a^		-	0.2 ^a^	-	0.1 ^a^
53	γ-Cadinene	1525	1512	0.3 ^a^	0.2 ^a^	-	0.2 ^a^	0.2 ^a^	0.3 ^a^	-	0.2 ^a^	0.2 ^a^
54	δ-Cadinene	1529	1519	0.5 ± 0.1	0.2 ^a^	0.6 ^a^	0.2 ^a^	0.1 ^a^	0.3 ^a^	0.1 ± 0.1	0.1 ^a^	0.1 ^a^
55	δ-Selinene	1545	1540	1.3 ± 0.4	0.8 ± 0.1	-	0.6 ± 0.1	0.7 ± 0.1	0.8 ± 0.2	0.6 ± 0.1	0.6 ^a^	0.7 ± 0.1
56	Selina-3,7(11)-diene	1550	1545	1.1 ± 0.1	0.3 ± 0.1	-	0.2 ^a^		0.4 ^a^	0.2 ^a^	0.2 ^a^	0.3 ^a^
57	*trans*-Nerolidol	1568	1562	0.7 ^a^	0.2 ^a^	0.2 ^a^	0.2 ^a^	0.1 ^a^	0.3 ^a^	0.1 ^a^	-	0.2 ^a^
58	Spathulenol	1585	1577	0.1 ^a^	-	-	-	-	-	-	-	-
59	Caryophyllene oxide	1593	1583	5.2 ± 0.2	1.1 ± 0.1	1.1 ^a^	0.9 ± 0.2		3.6 ^a^	0.5 ± 0.2	0.8 ± 0.1	0.8 ± 0.1
60	Viridiflorol	1607	1600	0.2 ^a^	-	-	0.3 ^a^		0.1 ^a^	-	-	0.3 ^a^
61	Guaiol	1619	1602	1.4 ± 0.1	0.2 ^a^	0.3 ^a^	0.1 ^a^		0.9 ^a^	-	0.2 ^a^	0.2 ^a^
62	Humulene-1,2-epoxide	1621	1615	-	-	-	0.2 ^a^	0.1 ^a^	-	-	-	0.2 ^a^
63	Caryophylla-4(12),8(13)-dien-5-ol	1628	1634	0.5 ^a^	0.1 ^a^	-	-		0.2 ^a^	-	0.1 ^a^	-
64	epi-γ-Eudesmol	1636	1625	0.2 ^a^	-	-	-	-	0.1 ^a^	-	-	-
65	Cubenol	1643	1643	0.5 ^a^	0.1 ^a^	-	0.1 ^a^	-	0.2 ^a^	-	0.1 ^a^	-
66	(*Z*)-14-Hydroxycaryophyllene	1667	1666	0.4 ^a^	-	-	-	0.1 ^a^	0.1 ^a^	-	0.1 ^a^	-
67	epi-α-Bisabolol	1681	1683	0.5 ± 0.1	-	-	0.1 ^a^	-	0.2 ^a^	-	-	-
68	β-Bisabolol	2041	-	0.1 ^a^	-	-	-	-	0.1 ^a^	-	-	-
69	Cannabidivarin (CBDV)	2193	2208	0.1 ^a^	0.1 ^a^	-	0.2 ^a^	-	0.2 ^a^	0.3 ^a^	0.2 ^a^	0.2 ^a^
70	Cannabichromene (CBC)	2772	-	-	-	-	-	-	-	-	-	-
71	Cannabidiol (CBD)	2779	-	0.6 ^a^	-	0.1 ^a^	-	-	0.1 ^a^	-	-	-
	Total area			94.7 ± 0.4	98.1 ± 0.9	97.4 ± 2.1	97.7 ± 0.7	98.0 ± 0.1	97.6 ± 0.3	98.4 ± 1.3	98.0 ± 0.1	98.2 ± 2.1

Experimental conditions as in Section 3.3 and Section 3.4. Percentage peak areas below 0.05% are indicated with -. ^a^ SD < 0.05. ^b^ LRI data from the NIST Chemistry WebBook, SRD 69, https://www.nist.gov.

**Table 3:** Minimum inhibitory concentration (MIC) of hemp EOs and common antibacterial drugs. Data are expressed as μg/mL.

Bacterial strain	EO1	EO2	EO3	EO4	EO5	EO6	EO7	EO8	EO9	EO10	EO11	EO13	EO14	EO15	EO16	EO17	Ampicillin	Ciprofloxacin
*Staphylococcus aureus* ATCC 6538	2	-	-	-	-	-	-	-	16	8	2	-	-	-	-	-	-	0.5
*Staphylococcus aureus* 18As *	-	-	-	16	32	-	-	-	-	16	-	-	-	-	-	-	-	16
*Staphylococcus epidermidi*s 18Bs *	4	-	-	16	-	16	-	-	1	8	-	16	-	-	-	-	-	0.5
*Staphylococcus aureus* 386 *	-	-	-	-	-	-	-	32	-	-	-	16	16	-	-	-	-	16
*Listeria monocytogenes* NCTC 10888	16	8	-	-	4	32	-	-	8	8	-	16	-	16	-	-	0.25	-
*Listeria monocytogene*s ATCC 13932	4	-	-	32	-	-	-	32	8	16	4	16	-	-	-	-	0.25	-
*Listeria monocytogenes* ATCC 5008	8	-	2	-	-	-	8	4	-	8	2	16	-	-	4	4	0.25	-
*Listeria monocytogenes* 70 *	4	-	-	-	32	32	2	16	-	-	4	-	-	-	-	-	2	-
*Listeria monocytogenes* 139 *	-	-	-	-	32	-	8	32	-	-	2	-	-	1	-	-	0.5	-
*Enterococcus faecalis* ATCC 29212	2	2	-	16	0.5	4	-	1	8	2	2	16	1	1	4	4		4
*Enterococcus hirae* ATCC 10541	2	-	-	32	-	16	4	-	4	16	16	16	32	-	-	-	-	8
*Enterococcus faecalis* V3 *	1	-	2	32	32	4	-	4	-	-	4	16	32	-	4	-	-	0.25
*Enterococcus faecalis* V4 *	2	-	-	-	-	32	8	16	16	16	2	-	-	-	-	-	-	16
*Enterococcus faecium* V5 *	-	-	1	-	16	4	8	8	1	16	2	2	32	16	-	-	-	8
*Enterococcus faecalis* V6 *	2	-	0.5	-	16	16	-	8	1	16	16	-	-	8	-	-	-	16
*Enterococcus faecium* EQ19 *	2	-	-	-	8	4	4	8	8	16	16	-	2	-	-	-	-	4
*Bacillus subtilis* ATCC 6633	2	8	2	8	8	16	-	16	4	-	8	-	1	2	-	4	2	-
*Bacillus cereus* EB 362	1	-	1	-	-	1	-	-	1	-	-	16	-	-	-	-	2	-
*Bacillus* 1 ˣ	2	8	-	-	8	1	-	-	4	8	16	16	-	4	-	-	1	-
*Bacillus* 2 ˣ	0.5	-	-	-	16	16	8	16	-	16	-	16	32	16	-	4	0.25	-
*Bacillus* 3 ˣ	-	-	2	8	-	16	8	32	-	-	16	-	16	16	4	-	1	-
*Bacillus* 4 ˣ	2	-	2	32	16	1	-	-	4	-	16	-	-	4	-	4	0.25	-
*Bacillus* 5 ˣ	2	-	2	-	-	4	-	32	4	-	-	16	-	4	-	-	2	-
*Bacillus* 6 ˣ	2	-	1	16	-	4	8	-	4	16	-	16	-	8	4	-	1	-
*Bacillus* 9 ˣ	-	-	2	8	8	32	-	-	8	-	-	-	-	4	-	-	1	-
*Bacillus* 10988 ˣ	2	-	-	-	-	4	2	32	4	-	16	16	-	8	-	4	2	-
*Bacillus* 18100 ˣ	0.5	-	-	-	-	16	-	-	4	-	-	-	-	-	-	-	0.5	-
*Bacillus* 18102 ˣ	2	-	2	-	-	16	-	-	4	-	16	16	-	16	-	-	0.25	-

* Bacteria isolated from food samples. ˣ Bacteria isolated from food environments.

**Table 4:** Minimum inhibitory concentration (MIC) of cannabidiol (CBD) and major terpenes of hemp EOs in comparison with common antibacterial drugs. Data are expressed as μg/mL.

Bacterial Strain	CBD	α-Pinene	β-Pinene	β-Myrcene	α-Terpinolene	β-Caryophyllene	Ampicillin	Ciprofloxacin
*Staphylococcus aureus* ATCC 6538	8	4	4	8	8	16	-	0.5
*Staphylococcus aureus* 18As *	32	16	32	8	32	32	-	16
*Staphylococcus epidermidi*s 18Bs *	16	8	4	16	16	32	-	0.5
*Staphylococcus aureus* 386 *	32	16	8	32	32	32	-	16
*Listeria monocytogenes* NCTC 10888	1	1	2	2	2	1	0.25	-
*Listeria monocytogene*s ATCC 13932	2	2	2	1	1	1	0.25	-
*Listeria monocytogenes* ATCC 5008	1	1	0.5	2	1	2	0.25	-
*Listeria monocytogenes* 70 *	4	2	2	2	4	4	2	-
*Listeria monocytogenes* 139 *	4	2	2	2	4	1	0.5	-
*Enterococcus faecalis* ATCC 29212	1	2	0.5	1	2	1		4
*Enterococcus hirae* ATCC10541	2	1	2	8	4	8	-	8
*Enterococcus faecalis* V3 *	1	1	1	4	1	2	-	0.25
*Enterococcus faecalis* V4 *	2	4	1	4	8	4	-	16
*Enterococcus faecium* V5 *	4	1	4	4	8	16	-	8
*Enterococcus faecalis* V6 *	4	1	2	8	16	1	-	16
*Enterococcus faecium* EQ19 *	1	4	2	1	2	4	-	4
*Bacillus subtilis* ATCC 6633	8	8	4	32	16	1	2	-
*Bacillus cereus* EB 362	8	2	1	2	4	8	2	-
*Bacillus* 1 ˣ	4	4	2	4	8	4	1	-
*Bacillus* 2 ˣ	8	8	4	4	16	16	0.25	-
*Bacillus* 3 ˣ	16	8	16	8	8	16	1	-
*Bacillus* 4 ˣ	8	16	16	16	16	8	0.25	-
*Bacillus* 5 ˣ	2	4	1	2	4	4	2	-
*Bacillus* 6 ˣ	4	2	1	4	4	8	1	-
*Bacillus* 9 ˣ	4	8	4	8	16	16	1	-
*Bacillus* 10988 ˣ	4	4	4	4	8	16	2	-
*Bacillus* 18100 ˣ	4	8	4	8	16	8	0.5	-
*Bacillus* 18102 ˣ	8	8	4	2	16	8	0.25	-

* Bacteria isolated from food samples. ˣ Bacteria isolated from food environments.

## Supplementary Materials

The following files are available online, Table S1: Inhibition diameters displayed by hemp EOs and common antibacterial drugs in the agar well disk diffusion assay. Data are expressed in mm.
