# Supplementary material for: Chemical Characterization and Evaluation of the Antibacterial Activity of Essential Oils from Fibre-Type Cannabis sativa L. (Hemp)
Source: Molecules. 2019 Jun 21;24(12):2302. doi: 10.3390/molecules24122302 (PMC6631254; doi:10.3390/molecules24122302)
Supplement: Supplementary file 1 [file molecules-24-02302-s001.pdf]

Chemical Characterization and Evaluation of the Antibacterial Activity of Essential Oils from Fibre-Type *Cannabis sativa* L. (Hemp)

Ramona Iseppi [1,†], Virginia Brighenti [1,†], Manuela Licata [2], Antonella Lambertini [1], Carla Sabia [1], Patrizia Messi [1], Federica Pellati [1,*] and Stefania Benvenuti [1]

[1] Department of Life Sciences, University of Modena and Reggio Emilia, Via G. Campi 103/287, 41125 Modena, Italy; ramona.iseppi@unimore.it (R.I.); virginia.brighenti@unimore.it (V.B.); anto.lambertini@gmail.com (A.L.); carla.sabia@unimore.it (C.S.); patrizia.messi@unimore.it (P.M.); stefania.benvenuti@unimore.it (S.B.)
[2] Department of Biomedical, Metabolical and Neural Sciences, University of Modena and Reggio Emilia, Via del Pozzo 71, 41124, Modena, Italy; manuela.licata@unimore.it (M.L.)
* Correspondence: federica.pellati@unimore.it; Tel.: +39-059-205-8565
† Both authors contributed equally to this work.

Supplementary Material

Table S1: Inhibition diameters displayed by hemp EOs and common antibacterial drugs in the agar well disk diffusion assay. Data are expressed in mm.

Table 1. Inhibition diameters displayed by hemp EOs and common antibacterial drugs in the agar well disk diffusion assay. Data are expressed in mm.

Bacterial strain	EO1	EO2	EO3	EO4	EO5	EO6	EO7	EO8	EO9	EO10	EO11	EO12	EO13	EO14	EO15	EO16	EO17	Ampicillin	Ciprofloxacin
Staphylococcus aureus ATCC 6538	24	0	0	0	0	10	12	12	15	18	24	0	0	10	0	0	0	-	23
Staphylococcus aureus 18As*	10	0	11	18	15	14	10	11	12	15	10	0	11	0	14	0	0	-	0
Staphylococcus epidermidis 18Bs*	21	0	13	20	13	18	13	11	25	22	13	0	16	0	11	0	0	-	32
Staphylococcus aureus 386*	11	13	12	12	13	11	11	15	12	13	10	0	16	19	0	10	13	-	0
Listeria monocytogenes NCTC 10888	19	16	13	13	23	16	13	11	17	21	13	9	15	11	16	13	13	14	-
Listeria monocytogenes ATCC 13932	21	13	11	15	13	14	15	15	18	16	20	6	16	11	8	11	11	12	-
Listeria monocytogenes ATCC 5008	18	0	17	14	11	14	28	24	14	21	24	0	16	11	0	17	15	14	--
Listeria monocytogenes 70*	21	11	0	11	15	15	16	18	13	11	21	6	13	11	9	7	13	0	-
Listeria monocytogenes 139*	14	14	0	13	15	14	13	15	12	13	25	0	11	11	30	0	10	12	-
Enterococcus faecalis ATCC 29212	28	22	14	18	28	22	20	30	19	26	27	0	18	30	30	16	16	-	29
Enterococcus hirae ATCC 10541	26	14	16	16	0	18	0	13	21	16	15	6	16	16	0	14	14	-	15
Enterococcus faecalis V3*	32	14	16	15	16	24	16	25	14	14	21	0	14	15	11	16	11	-	40
Enterococcus faecalis V4*	26	0	0	0	14	16	18	18	15	18	25	0	12	0	0	0	0	-	0
Enterococcus faecium V5*	11	0	21	0	18	24	13	20	24	18	27	0	24	16	15	0	0	-	19
Enterococcus faecalis V6*	26	13	16	11	18	18	21	22	25	16	16	0	14	13	18	14	11	-	0
Enterococcus faecium EQ19*	28	11	11	13	22	24	10	20	19	18	18	0	9	23	11	11	13	-	24
Bacillus subtilis ATCC 6633	26	16	17	27	21	17	0	18	21	0	19	14	12	32	26	14	17	34	-
Bacillus cereus EB 362	29	14	21	12	14	32	0	0	30	0	11	9	17	11	14	14	13	34	-
Bacillus 1[x]	30	17	11	14	21	30	18	14	23	21	17	0	15	0	21	11	10	38	-
Bacillus 2[x]	35	11	13	14	18	19	18	18	11	17	13	9	16	15	18	13	17	29	-
Bacillus 3[x]	12	9	18	23	11	19	14	15	11	11	18	0	14	18	15	15	11	30	-
Bacillus 4[x]	29	11	16	16	18	35	0	11	25	14	16	6	11	9	22	14	16	37	-
Bacillus 5[x]	27	6	18	10	14	25	16	15	22	11	11	0	18	11	21	11	13	29	-
Bacillus 6[x]	30	13	21	18	11	21	11	13	21	18	14	0	15	0	17	16	14	38	-
Bacillus 9[x]	11	12	16	23	22	15	24	14	17	0	12	0	13	12	20	11	11	34	-
Bacillus 10988[x]	32	11	11	13	12	23	0	16	20	0	18	9	16	11	18	11	18	30	-
Bacillus 18100[x]	35	12	14	13	0	20	14	10	22	11	13	11	13	13	11	11	0	34	-
Bacillus 18102[x]	32	0	15	9	8	18	11	11	24	9	15	0	16	11	15	9	0	40	-

* Bacteria isolated from food samples. [x] Bacteria isolated from food environments.
